# Picolinamide Functionalization on Carbon Nitride Edges for Enhanced Charge Separation and Photocatalytic Hydrogen Evolution

**DOI:** 10.3390/nano15050361

**Published:** 2025-02-26

**Authors:** Peiru Li, Siyuan Guo, Yunan Liu, Yanhong Lin, Tengfeng Xie

**Affiliations:** College of Chemistry, Jilin University, Changchun 130012, China; lipr24@mails.jlu.edu.cn (P.L.);

**Keywords:** carbon nitride, picolinamide doping, photocatalytic H_2_ production

## Abstract

The periodical distribution of N and C atoms in carbon nitride (CN) not only results in localized electrons in each tri-s-triazine unit, but oxidation and reduction sites are in close contact spatially, resulting in severe carrier recombination. Herein, the hydrothermal method was first employed to synthesize carbon nitride (HCN), and then picolinamide (Pic) molecules were introduced at the edge of the carbon nitride so that the photo-generated electrons of the whole structure of the carbon nitride system were transferred from the center to the edge, which effectively promoted the separation of photo-generated carriers and inhibited the recombination of carriers in the structure. The introduced picolinamide not only changed the π-conjugated structure of the entire system but also acted as an electron-withdrawing group to promote charge transfer. The photocatalytic hydrogen evolution rate (HER) of the optimized HCN-Pic-1:1 sample could reach 918.03 μmolg^−1^ h^−1^, which was 11.8 times higher than that of the HCN, and the performance also improved.

## 1. Introduction

With the development of technology and the improvement of people’s living standards, the demand for energy by humans is also increasing day by day. However, the extensive use of fossil fuels not only brings serious environmental pollution problems to human beings, the excessive exploitation of fossil fuels can ultimately lead to energy depletion, which has attracted widespread attention [1,2,3,4]. Therefore, finding a clean and renewable energy source is an effective way to solve these problems. As a new type of energy, hydrogen energy has only water as the product after combustion, generating high heat during combustion [5,6,7], and can react with carbon-containing substances, such as CO_2_, to produce fuels such as methanol, which has attracted widespread attention. A photocatalytic reaction serves as a crucial means for achieving the conversion from solar energy to chemical energy. Among them, photocatalytic water splitting for hydrogen production stands as one of the most prevalent photocatalytic reduction reactions [8,9,10]. In recent years, the types of photo-catalysts have also increased, such as TiO_2_ [11], MOF [12], various sulfides [13], g-C_3_N_4_ [14,15,16], and so on. Graphitic carbon nitride has a wide range of applications due to its simple synthesis method [17], suitable conduction and valence band positions, and good thermal stability [14,18,19]. However, primal carbon nitride also has disadvantages, such as high carrier recombination efficiency and a large number of terminal amino groups, which limits its application in photocatalytic hydrogen evolution [20,21,22]. Therefore, in order to improve the photocatalytic hydrogen production performance of carbon nitride, we should improve the performance from the perspective of promoting the separation and migration of photo-generated carriers.

The skeleton structure of CN is a network composed of triazines or tri-s-triazines connected to tertiary amines. The highly symmetrical structure of carbon nitride not only results in local electrons in each tri-triazine unit but also tightly contacts the oxidation and reduction sites in space, leading to severe carrier recombination [23,24]. Inducing n→π* electronic transitions in carbon nitride represents a promising strategy for enhancing its photocatalytic performance. However, the inherent structural constraints of carbon nitride, characterized by its high symmetry and planar configuration, inherently prohibit such electronic transitions, limiting the exploration of this approach. Unlike π→π* transitions, n→π* transitions typically exhibit a lower transition probability and are classified as symmetry-forbidden transitions. This restriction arises from the symmetry mismatch between the n orbital and the π orbital, which hinders efficient electron transfer between these orbitals. To address this limitation, introducing structural distortions into carbon nitride emerges as a viable solution to facilitate n→π* electronic transitions. By strategically relocating lone electron pairs into a planar arrangement, transitions to the π* orbital can be enabled. The development of a precise and controllable method to modulate n→π* electronic transitions in carbon nitride is of paramount importance for advancing its photocatalytic applications [25]. Therefore, the improvement of carrier separation efficiency is the key factor determining the photocatalytic hydrogen evolution efficiency [26,27].

Previous studies have shown that introducing structurally matched aromatic functional groups into CN framework structures [28,29,30,31] can effectively regulate the physicochemical properties of CN-based materials, such as light absorption, hydrophilicity, conductivity, etc. Due to their structural similarity, incorporating benzene rings into the framework of CN is a common strategy for regulating the structure of CN [32,33]. The introduction of benzene rings can facilitate the migration of π electrons in CN materials, thereby suppressing the recombination of electron hole pairs without significantly affecting the structure of CN. Research has shown that by adjusting experimental parameters, such as the type of precursor [34], reaction temperature [35], and pH [36], the position of the benzene ring can be controlled. Even with the introduction of π-conjugated aromatic molecules, there is still some recombination of photo-generated electrons and holes in the CN structure, which reduces the efficiency of photocatalytic hydrogen evolution. Some studies have shown that introducing electron-withdrawing groups or organic small molecules with electron-withdrawing properties at the edges of carbon nitride promotes the transfer of electrons from the CN basic unit to the electron-withdrawing small molecules at the edges of the entire structure [37,38]. This method not only promotes charge transfer but also changes the electronic hybridization structure of the entire structure and reduces the recombination of photogenerated electrons and holes, thereby improving the efficiency of photocatalytic hydrogen production. In summary, the introduction of picolinamide may induce n→π* electronic transitions by disrupting the highly symmetric structure of carbon nitride, thereby enhancing the generation and migration efficiency of photo-generated charge carriers. Additionally, the nitrogen atoms and conjugated structure within picolinamide can serve as electron acceptors, improving the separation efficiency of photo-generated electrons and holes in carbon nitride. This reduces the recombination of electron–hole pairs and enhances photocatalytic activity. Furthermore, the conjugated structure and chemical stability of picolinamide can improve the overall stability of carbon nitride, extending its lifespan in photocatalytic reactions.

Herein, we used urea and melamine as precursors to form supramolecular precursors through hydrothermal treatment [39]. After drying, they underwent thermal polymerization with picolinamide (Pic) to graft picolinamide molecules as electrons withdrawing groups onto the edges of the CN skeleton. In the CN (Pic-CN) sample grafted with pyridine amide molecules at the edge, directional charge transfer from the center to the edge was achieved while also changing the electronic hybridization of the entire structure. The charge separation efficiency was significantly improved, ultimately enhancing the photocatalytic HER performance.

## 2. Materials and Methods

### 2.1. Chemicals

Urea (AR) was purchased from Tianjin Yongda Chemical Reagent Co., Ltd. (Tianjin, China). Melamine (AR) was purchased from Shanghai Aladdin Biochemical Technology Co., Ltd. (Shanghai, China). Picolinamide was purchased from Aladdin. Triethanolamine and H_2_PtCl_6_ (AR) were purchased from Sinopharm. The nitrogen gas was normal nitrogen and was purchased from Changchun Juyang Gas Co., Ltd. (Changchun, China). All experimental drugs were purchased directly and were not purified before use. Distilled water was used in all experiments.

### 2.2. Synthesis of PCN

In a typical synthesis, 5 g of melamine was calcined at 550 °C in air for 4 h at a heating rate of 2 °C/min, and the resulting buff powder was collected.

### 2.3. Synthesis of HCN

The HCN samples were prepared by using 1.5 g of melamine and 2.4 g of urea as precursors in 40 mL of deionized water under continuous stirring for 30 min at room temperature to ensure homogeneity. The resulting mixture was then transferred into a reaction kettle and subjected to thermal treatment in an oven at 180 °C for 12 h to facilitate the polymerization process. After cooling, the solid product was collected via centrifugation and subsequently dried in a vacuum drying oven at 60 °C for 24 h to remove residual moisture. The dried material was calcined at 550 °C in air for 4 h at a heating rate of 2 °C/min, and the resulting buff powder was collected.

### 2.4. Synthesis of the Edge-Grafted Picolinamide HCN Sample (HCN-Pic-x)

The edge-grafted picolinamide HCN samples were prepared by mixing 2 g of HCN with a different amount of picolinamide (Pic). The solids were calcined at 550 °C in air for 4 h at a heating rate of 2 °C/min. The final samples were denoted as HCN-Pic-x, where x represents HCN and Pic with different mass ratios (x = 5:1, 2:1, 1:1, and 1:2), and the resulting brown powder was collected.

### 2.5. Characterization

X-ray diffraction (XRD) was carried out on a Rigaku D/Max—2550 using Cu Kα radiation. The scan was performed at a rate of 10°/min from 10° to 80°. The accelerating voltage and current were 40 kV and 80 mA. Scanning electron microscopy (SEM) images were analyzed by Hitachi S-4800 (Tokyo, Japan). Transmission electron microscopy (TEM) images were obtained with a JEOL 2100PLUS electron microscope (Akishima, Japan) with an accelerating voltage of 200 kV. The Fourier-transform infrared (FTIR) spectra were measured on a Thermo Fisher Scientific system (Nicolet 6700, Waltham, MA, USA) in a KBr pellet, scanning from 4000 to 400 cm^−1^ at room temperature. Raman spectroscopy analysis was carried out on a Thermo DXRxi in the United States. X-ray photoelectron spectroscopy (XPS) measurements were conducted on an ESCALAB 250Xi electron spectrometer (Thermo Fisher Scientific, Waltham, MA, USA) with an Al Kα (1286.6 eV) source. All binding energies were corrected using the C 1s peak at 284.6 eV for binding energy calibration. The BET surface areas were calculated from nitrogen adsorption–desorption isotherms at 77 K using a Micromeritics ASAP 2420 surface area analyzer, and the samples were degassed at 393 K for 12 h. UV-Vis diffuse reflectance spectra were measured using a UV/Vis near-infrared (UV/Vis-NIR) spectrophotometer (with BaSO_4_ as the background, Shimadzu UV-3600, 300–800 nm). Photoluminescence (PL) and time-resolved photoluminescence (TR-PL) were measured using an Edinburgh Instruments FLS920 photoluminescence spectrometer, excited at a wavelength of 365 nm. The transient photocurrent responses and electrochemical impedance spectroscopy (EIS) were measured using a CHI 600E electrochemical workstation equipped with a standard three-electrode cell system. Within this system, the working electrode was an indium tin oxide (ITO) glass substrate with the previously mentioned samples deposited on it. Meanwhile, a platinum wire was utilized as the counter electrode, and an Ag/AgCl electrode was adopted as the reference electrode. A 1 mol/L Na_2_SO_4_ solution was chosen as the electrolyte.

### 2.6. Photocatalytic HER Experiments

A 10 mg sample of the photo-catalyst was accurately weighed and transferred to a beaker. Subsequently, 30 mL of a 10% triethanolamine (TEOA) solution was added to the beaker. The mixture was then subjected to ultrasonic stirring for 10 min to ensure uniform dispersion of the photocatalyst in the TEOA solution. The resulting suspension was then transferred into a 100 mL quartz reactor, followed by the addition of 70 mL of TEOA solution and 83 μL (3 wt%) of H_2_PtCl_6_ solution as the co-catalyst. The reactor was sealed and placed under dark conditions. Nitrogen gas was introduced for 30 min to remove air from the reactor. The sealed reactor was then placed in a reaction cell equipped with a quartz plate illumination window and subjected to photocatalytic hydrogen evolution under irradiation from a 300 W xenon lamp. Hydrogen production was monitored using a thermal conductivity detector (TCD) in gas chromatography (Shimadzu, GC 2014C TCD, Kyoto, Japan), with high-purity nitrogen as the carrier gas and a 5A molecular sieve column. To ensure experimental reproducibility, each photocatalytic test was performed in triplicate under identical conditions.

### 2.7. Characterization of Photo-Generated Charge Behavior

The surface photovoltage (SPV) testing system was independently built by the laboratory, and the entire system included a 500 W xenon lamp (Changtuo Chemical Reagent Co., Ltd., CHF-XM500, Beijing, China), a monochromator (ZLolix, SBP500, Beijing, China), a lock-in amplifier (SR830, Stanford Research System, Inc., Sunnyvale, CA, USA), chopper (SR540, Stanford Research System, Inc., Sunnyvale, CA, USA), sample cell, and computer. The intensity of monochromatic light could be adjusted by controlling the intensity of the xenon lamp. The computer controlled the wavelength and frequency of the modulated light by controlling the chopper and the monochromator. The obtained signals were collected by a lock-in amplifier. The test structure of the sample was “FTO-sample-mica-FTO”, with a scanning wavelength range of 300–800 nm. Transient surface photovoltage (TPV) was excited based on laser pulses. The signal of the sample cell was first amplified by a preamplifier (5003, Brookdeal Electronics, Wokingham, UK) and finally recorded and collected by a 500 MHz oscilloscope (TDS5054, Tektronix, Beaverton, OR, USA). The sample assembly in the sample pool was the same as the SPV test, and it was also an “FTO-sample-mica-FTO” structure.

## 3. Results and Discussion

### 3.1. Structure and Morphologies

**Figure 1:**
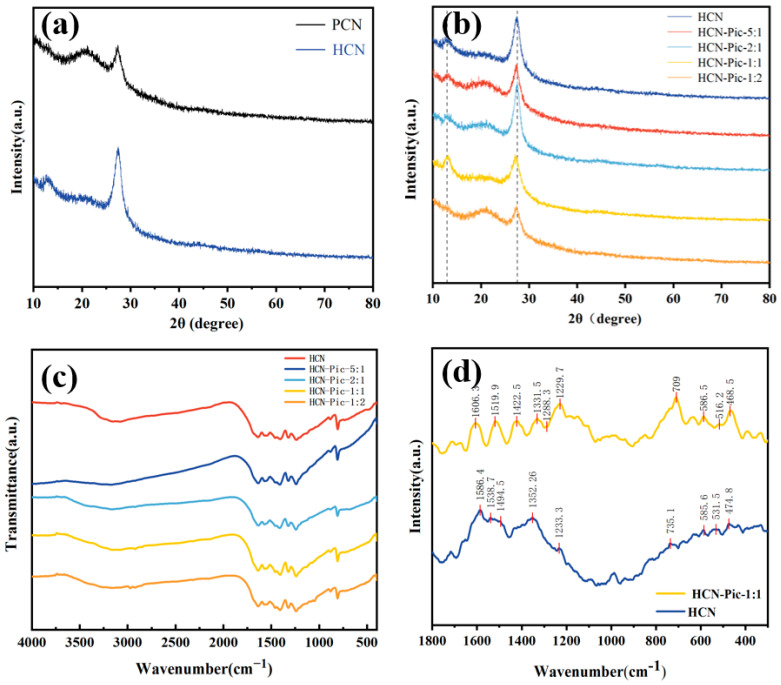
(**a**) XRD pattern of PCN and HCN; (**b**) XRD pattern of HCN and different additional amounts of Pic; (**c**) FT-IR spectra of HCN and different additional amounts of Pic; (**d**) Raman spectra of HCN and HCN-Pic-1:1.

As shown in Figure 1a, the crystallinity of the HCN sample was significantly higher than that of PCN. Supramolecular self-assembly and pre-polymerization were carried out by hydrothermal treatment to form supramolecular precursors, which improved the crystallinity of carbon nitride. This method did not require the use of any expensive external hard or soft templates, and the precursor monomers formed self-assembled complexes through weak interactions, resulting in relatively uniform structures in various solvent environments. In the subsequent thermal polymerization process, the self-assembled complexes could be transformed into CN with high specific surface areas and unique morphologies. As shown in Figure 1a, HCN exhibited two characteristic peaks of carbon nitride at 12.7° and 27.56°, corresponding to the in-plane structure (100) and interlayer stacking (002) of carbon nitride, respectively [40,41]. The analogous diffraction patterns of HCN-Pic samples indicated that the addition of picolinamide to the carbon nitride end did not alter the overall crystal structure of CN. In addition, as shown in Figure 1b, it can be observed that with the addition of picolinamide, the picolinamide reduced the crystallinity of the whole carbon nitride structure, and the (002) crystal surface moved from 27.38° to 27.50°, which also proves that the addition of picolinamide reduced the distance between interlayer stacking [42].

Molecular structure characterization was analyzed based on the FT-IR spectra presented in Figure 1c. Comparing the FTIR spectrum of HCN with the spectra with different Pic ratios (HCN-Pic-5:1, HCN-Pic-2:1, HCN-Pic-1:1, and HCN-Pic-1:2) revealed characteristic vibrational bands at 810 cm^−1^, 1500–1600 cm^−1^, 1200–1700 cm^−1^, and 3000–3400 cm^−1^, corresponding to the vibration of the tris-triazine ring, C-C stretching vibration of the pyridine molecular ring, stretching vibration of the aromatic CN heterocyclic ring, and N-H stretching vibration of the terminal amine group (-NH_2_). The preservation of these characteristic peaks across all samples indicated that the incorporation of picolinamide did not modify the fundamental structure of the CN framework. Instead, the spectral evidence suggests that picolinamide molecules were predominantly attached to the edges of HCN fragments, as supported by previous studies [43]. Through photocatalytic hydrogen production tests, we determined that HCN-Pic-1:1 was the best sample, which will be described in Section 3.2. Subsequent tests were all carried out on HCN and HCN-Pic-1:1 samples.

As shown in Figure 1d, the molecular structures of the samples were also examined using Raman spectroscopy. The HCN exhibited heterocyclic peaks corresponding to CN at 1586.4, 1538.7, 1494.5, 1352.56, 1233.3, 735.1, 585.6, 531.5, and 474.8 cm^−1^ [44]. Additionally, HCN-Pic-1: 1 showed that a new C-C peak appeared at 1606.3 cm^−1^, and the peaks at 1229.7 and 1288.3 cm^−1^ corresponded to the C-H bond peaks [45]. This also indicated that the picolinamide was well doped into the CN structure without causing any structural changes.

**Figure 2:**
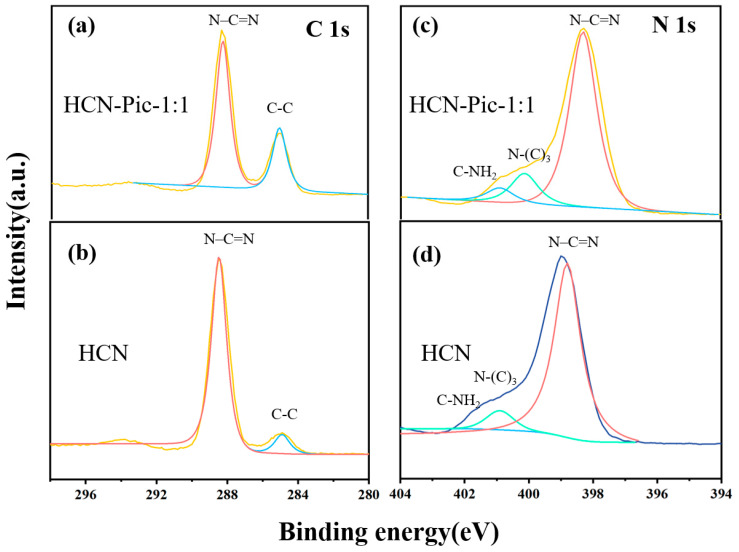
(**a**,**b**) C 1s XPS spectra; (**c**,**d**) N 1s XPS spectra of HCN and HCN-Pic-1:1.

Furthermore, in the XPS C 1s spectrum of the HCN and HCN:Pic-1:1 samples presented in Figure 2a,b, the two samples had two obvious peaks at 284.6 and 288.2 eV in the C 1s spectrum, corresponding to graphite carbon (C-C/C=C) and sp_2_ hybrid carbon (N–C=N), respectively [46]. In Figure 2c,d, the N 1s spectrum of the HCN-Pic-1:1 sample could be divided into three peaks centered at 398.3, 400.2, and 400.9 eV, which were respectively attributed to sp_2_ hybrid nitrogen (N-C=N), tertiary nitrogen (N-C_3_), and terminal amino (C-NH_2_). However, for the HCN-Pic-1:1 sample, we found that the peaks from (N-C_3_) and (C-NH_2_) were significantly shifted to the high binding energy, which indicates that the introduction of the electron-withdrawing group picolinamide molecules into the CN skeleton reduced the electron density around the nitrogen atom, and the entire structure electron migrated from the skeleton to the edge. In addition, the XPS C 1s spectra also showed that the percentage of carbon atoms increased after the introduction of picolinamide molecules [47]. As shown in Table 1, the percentage content of oxygen increased from 3.08% to 4.58%. It can be seen on the surface that picolinamide molecules were doped into the end edge of carbon nitride.

**Figure 3:**
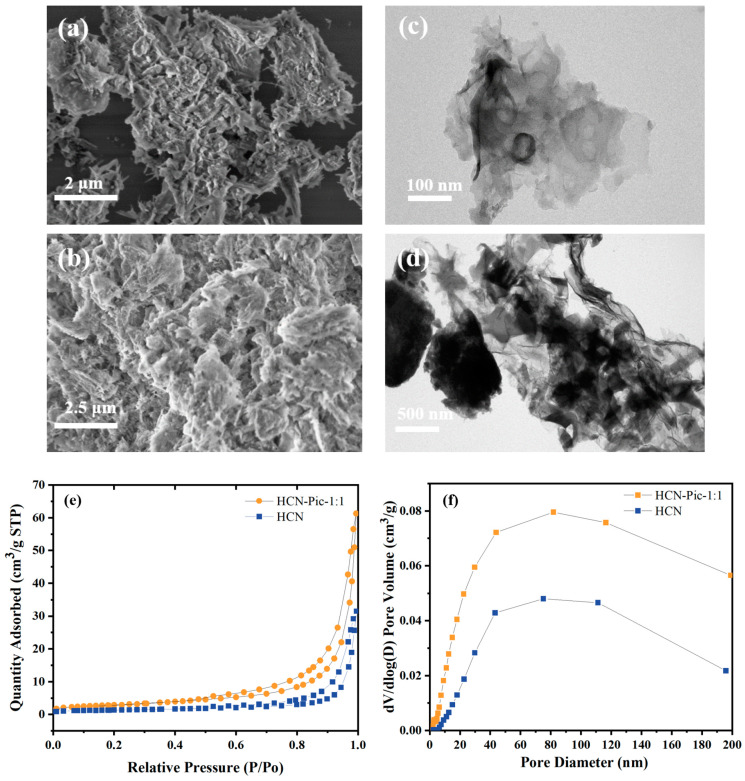
(**a**) SEM images of HCN; (**b**) SEM images of HCN-Pic-1:1; (**c**) TEM images of HCN; (**d**) TEM images of HCN-Pic-1:1; (**e**) nitrogen adsorption–desorption isotherm of HCN and HCN-Pic-1:1; (**f**) pore size distributions of HCN and HCN-Pic-1:1.

The morphology and microstructure of the samples were systematically characterized using scanning electron microscopy (SEM) and transmission electron microscopy (TEM). As depicted in Figure 3a,b, the introduction of picolinamide molecules resulted in a more porous and looser surface structure in the HCN-Pic-1:1 sample, compared to pristine HCN. Furthermore, the TEM images in Figure 3c,d revealed that with the addition of picolinamide, the edge of the CN structure bent. Based on the results shown in Figure 3e, nitrogen adsorption–desorption measurements demonstrated a significant increase in specific surface area from 4.6027 m^2^/g for HCN to 10.3980 m^2^/g for the HCN-Pic-1:1 sample. Also, based on the results presented in Figure 3f, it was indicated that the HCN-Pic-1:1 composite possessed larger pores, compared to the unmodified HCN. These structural modifications, including the increased specific surface area and optimized pore structure, are believed to enhance water and gas adsorption capacity, while the formation of thinner, sheet-like structures provides more exposed active sites, ultimately contributing to the improved photocatalytic hydrogen evolution performance.

### 3.2. Photocatalytic HER Performance

**Figure 4:**
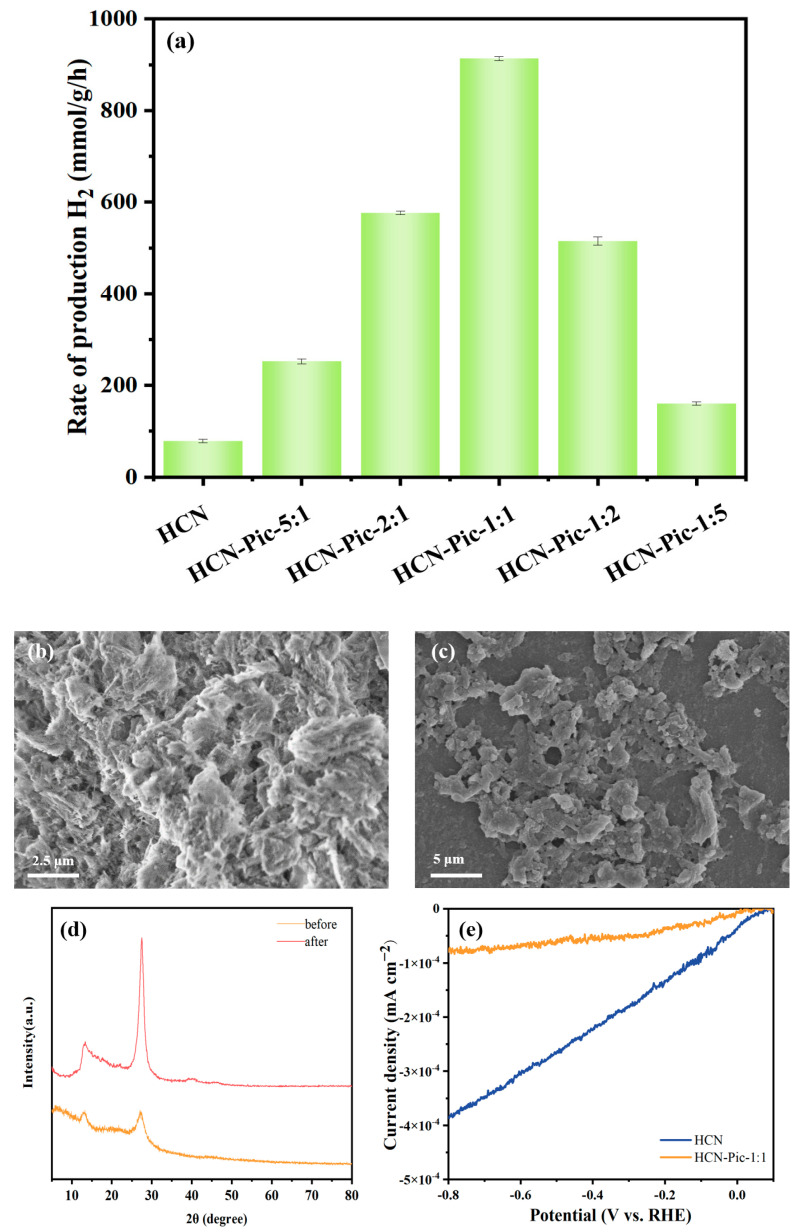
(**a**) Photocatalytic hydrogen production efficiency of samples with different amounts of picolinamide; (**b**) SEM images of HCN-Pic-1:1; (**c**) SEM images of HCN-Pic-1:1 after reaction; (**d**) XRD patterns of the HCN-Pic-1:1 sample before and after HER cycles; (**e**) electrocatalytic HERs of HCN-Pic-1:1 and HCN.

The photocatalytic HER performance of the sample was measured in a quartz reactor using triethanolamine as a sacrificial agent and 3wt% H_2_PtCl_6_ as a co-catalyst. The photocatalytic hydrogen production activity of HCN-Pic was determined under the irradiation of a 300 W Xe lamp and a 420 nm cut-off filter with different amounts of picolinamide added. Figure 4a reveals that as the amount of picolinamide added increased, the hydrogen production activity of HCN-Pic samples significantly increased. Among them, the HCN-Pic-1:1 sample had the highest photocatalytic hydrogen production rate of 918.03 μmol g^−1^ h^−1^, which is about 11.8 times that of the original nitrogen-doped carbon HCN (78.06 μmol g^−1^ h^−1^). From Figure 4b,c, which shows SEM images of the samples before and after the photocatalytic hydrogen production reaction, it can be seen that the structure of the HCN-Pic-1:1 sample remained basically unchanged. After the photocatalytic hydrogen production reaction, XRD tests (Figure 4d) were conducted on the HCN-Pic-1:1 samples before and after the reaction, which also showed that the samples had good recyclability, and diffraction peaks of Pt appeared at 39.8° and 46.2°. We conducted electro-catalytic HER tests on the HCN and HCN-Pic-1:1 samples. As shown in Figure 4e, the results showed that the current density of the HCN-Pic-1:1 sample increased. However, the current densities of both samples were very small, indicating weak electro-catalytic HER performance.

### 3.3. Mechanism of Photocatalytic Activity Enhancement

**Figure 5:**
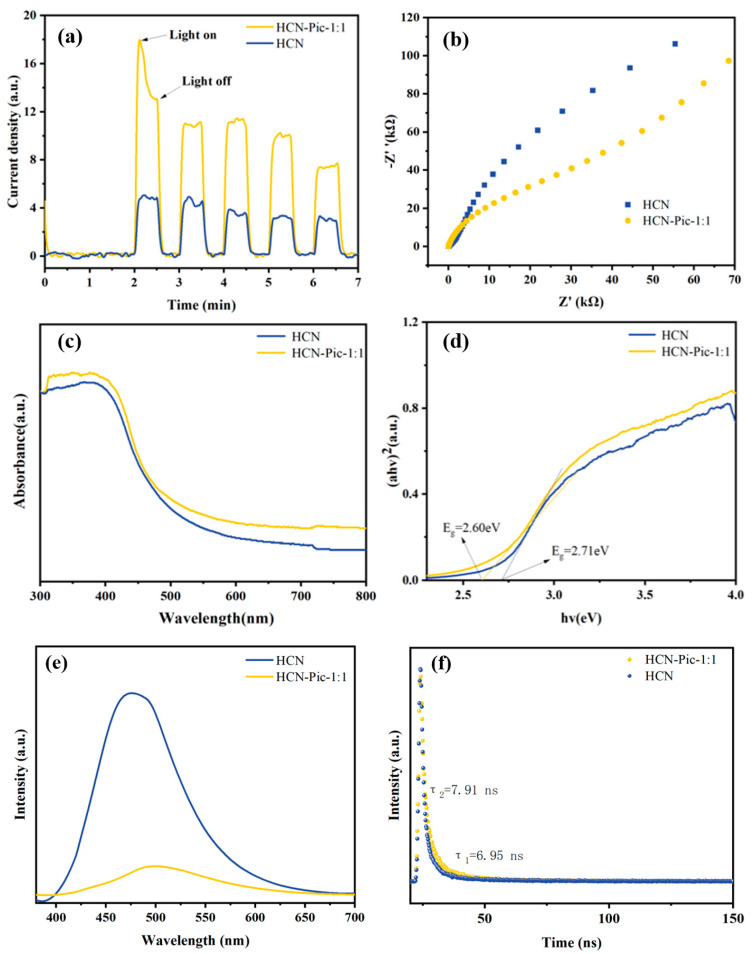
(**a**) Transient photocurrent curves under the visible light; (**b**) EIS Nyquist plots; (**c**) UV–vis DRS; (**d**) plots of (αhν)^2^ versus (hν); (**e**) PL spectra; (**f**) TR-PL decay curves of HCN and HCN-Pic-1:1 samples.

The key to improving the photocatalytic hydrogen production rate is to improve the migration and separation efficiencies of photogenerated carriers while inhibiting their recombination, which can be reflected by photovoltage, electrochemical, and photo-electrochemical experiments. The photocurrent responses of the HCN and HCN-Pic-1:1 samples were evaluated (Figure 5a), showing that the HCN-Pic-1:1 composite achieved a 3.57-times enhancement in the photocurrent, compared to HCN. This result clearly indicated a substantial improvement in the separation and transfer efficiency of photo-induced charge carriers. It showed that the transition of electrons from n→π* orbitals significantly promoted the separation of electrons and holes, thus improving the photocatalytic performance. In addition, as shown in Figure 5b by electrochemical impedance spectroscopy (EIS), the EIS Nyquist diagram on the HCN-Pic-1:1 electrode showed that compared with HCN, the resistance of the HCN-Pic-1:1 electrode was lower, and the interface charge transfer ability was faster. This result is consistent with the photocurrent measurement [48]. The optical absorption properties of the HCN samples were investigated using UV-Vis absorption spectroscopy, as shown in Figure 5c. Compared with HCN, the visible light absorption of the HCN-Pic sample at 420 nm was significantly improved. By introducing picolinamide molecules, the utilization of light was improved, thus enhancing the ultraviolet absorption and visible light capture [49].

The optical properties of the synthesized samples were systematically investigated using UV/Vis spectrometry. The absorption spectra were converted into Tauc plots using the equation (αhν)^2^ = A(hv − E_g_), which is specifically applicable for direct bandgap semiconductors. By extrapolating the linear region of these plots, the bandgap energies of the synthesized samples were determined. In this equation, A is a constant related to the material, while E_g_ denotes the bandgap energy. By extrapolating the linear portion of the Tauc plot to intersect the abscissa, the energy value at the intersection point was determined as the band gap (E_g_) of the semiconductor material. Based on the comparison of (αhν)^2^ versus hν shown in Figure 5d, the bandgap energies of HCN and HCN-Pic-1:1 were calculated to be 2.71 eV and 2.60 eV, respectively. This notable reduction in bandgap energy following the incorporation of picolinamide suggests an effective modification of the electronic structure, potentially enhancing the material’s visible light absorption capability.

The photoluminescence (PL) and time-resolved photoluminescence (TR-PL) further revealed the separation and transfer efficiency of charge carriers. As shown in Figure 5e, when the samples were excited by 365 nm of visible light, for HCN-Pic-1:1, the fluorescence intensity of the sample was significantly lower than that of HCN, and the decrease in PL intensity can indicate that the recombination efficiency of carriers is reduced, which also indicates that there is faster carrier migration efficiency and that more photo-generated electrons and holes participate in the reaction. Picolinamide as an electron-withdrawing group can capture electrons, while carbon nitride as an electron donor can promote the carrier migration rate. It can be seen from Figure 5f that under the same excitation wavelength, the lifetime of photo-generated carriers was studied by time-resolved photoluminescence spectroscopy. The average (τ) PL lifetime of HCN-Pic-1:1 (τ_2_ = 7.91 ns) increased, compared with the HCN (τ_1_ = 6.95 ns) [48,50,51].

**Figure 6:**
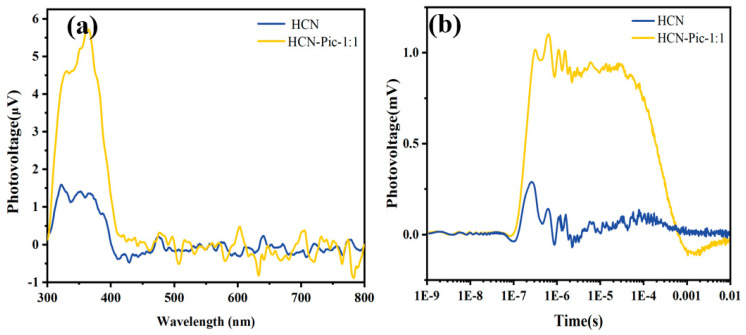
(**a**) Surface photovoltage (SPV) spectra; (**b**) transient-state surface photovoltage (TPV) spectra of HCN and HCN-Pic-1:1 samples.

To further investigate the effect of picolinamide molecule addition on the photo-generated charge mobility within the entire structure, we measured the surface photovoltage (SPV) [52], as shown in Figure 6a. The results showed that the photovoltage signals had a significant enhancement. This observation indicates that the electron-withdrawing picolinamide group improved the separation efficiency of photo-generated carriers. In order to further reveal the enhancement of the separation ability of photo-generated carriers, we also provided the TPV spectrum under a 355 nm pulsed laser, as shown in Figure 6b. The variation in the signal intensity of the TPV spectrum mirrored that of the steady-state photovoltage (SPV) spectrum [53]. The results demonstrated that, compared to HCN, the introduction of picolinamide promoted a rapid migration of electrons from the carbon nitride ring to the CN end, improving the separation efficiency of photo-generated carriers and, consequently, enhancing the photocatalytic performance of the entire catalyst.

## 4. Conclusions

In summary, the specific surface area of carbon nitride was increased by hydrothermal synthesis, and then an edgewise modified carbon nitride structure was formed by introducing electron-absorbing group picolinamide and carbon nitride structure by calcination. Through photo-physical, electrochemical, and photo-electrochemical tests, it can be shown that the addition of picolinamide can change the hybrid structure of the whole system, and at the same time, a photo-generated charge can quickly migrate from the CN structure to the terminal picolinamide group after excitation. Compared with HCN, the photocatalytic hydrogen production performance reached 918.03 μmolg^−1^ h^−1^, which was 11.8 times that of HCN. We believe that our work contributes to the interaction of small organic molecules grafted on the surface of photo-catalysts and provides guidance for the design and modification of CN-based photo-catalysts.

## Figures and Tables

**Table 1:** Atom contents derived from XPS analysis.

Sample	C%	N%	O%
HCN	42.58	54.34	3.08
HCN-Pic-1:1	47.43	47.98	4.58

## Data Availability

Data will be made available upon request.

## Abbreviations

The following abbreviations are used in this manuscript:

CN	carbon nitride
HCN	hydrothermal method to synthesize carbon nitride
Pic	picolinamide
BCN	bulk carbon nitride
HCN	hydrothermal carbon nitride
XRD	X-ray diffraction
SEM	scanning electron microscopy
TEM	transmission electron microscopy
FTIR	Fourier-transform infrared
XPS	X-ray photoelectron spectroscopy
PL	photoluminescence emission
HER	photocatalytic hydrogen production rate
SPV	surface photovoltage
TPV	transient surface photovoltage
